# Editorial: Cardiovascular Imaging Case Reports 2025: Emphasizing Uncommon Clinical Scenarios

**DOI:** 10.3389/fcvm.2026.2003404

**Published:** 2026-10-02

**Authors:** Antonios Karanasos, Grigorios Korosoglou

**Affiliations:** 1 Department of Cardiology, University of Patras, Patras, Greece; 2 Departments of Cardiology & Vascular Medicine, GRN Hospital Weinheim, Weinheim, Germany; 3 Departments of Cardiology & Vascular Medicine, GRN Hospital Eberbach, Eberbach, Germany; 4 Cardiac Imaging Center Weinheim, Hector Foundation, Weinheim, Germany

**Keywords:** cardiac computed tomography (CCT), cardiac magnetic resonance (CMR), cardiomyopathies, echocardiography, ischemic heart disease, multimodality cardiovascular imaging

Cardiology and vascular medicine continue to rapidly evolve in terms of percutaneous therapeutic capabilities for our patients, yet accurate diagnosis and effective management of complex cardiovascular diseases remain major clinical challenges that require increasingly advanced diagnostic tools. Multimodality imaging is central to this process, offering essential insights into cardiac anatomy, perfusion, viability, function, and tissue characterization. Technological advances in recent decades have expanded clinicians’ ability to assess complex clinical problems. Available modalities range from echocardiography, the established first-line imaging technique, to cardiac magnetic resonance (CMR) and computed tomography (CT), each with distinct strengths and limitations. Therefore, a multimodality imaging approach that combines their complementary advantages is often needed in clinical practice. The present collection presents real-world cases in which cardiovascular imaging, used alone or in combination, helped resolve diagnostic uncertainty, characterize pathology precisely, and guide optimal treatment strategies.

## Non-invasive imaging: from serial echocardiography to the integrative approach of multimodality imaging

Echocardiography was central to assessing cardiac dimensions and function in a 79-year-old woman with paroxysmal atrial fibrillation (Korosoglou et al.). The patient presented with signs of congestive heart failure, and echocardiography revealed new-onset tricuspid annular dilatation with torrential tricuspid regurgitation. These findings guided management and led to deferral of minimally invasive tricuspid valve clipping. After successful electrical cardioversion, reverse right atrial remodeling occurred, with resolution of atrial enlargement and tricuspid regurgitation. Clinical symptoms also improved within a few weeks. To maintain sinus rhythm, the patient underwent cryoablation, and her subsequent clinical course was uneventful.

The central role of non-invasive cardiovascular imaging for the diagnosis of microvascular dysfunction in a patient with cardiac amyloidosis is highlighted by Yuqian et al. Cardiac amyloidosis (CA) is a rare but increasingly recognized cause of myocardial storage disease causing heart failure (1–3). In a patient with light-chain (AL) cardiac amyloidosis and exertional angina, contrast adenosine stress echocardiography with Doppler-derived coronary flow velocity reserve (CFVR) showed impaired myocardial perfusion. These findings were consistent with CMR and laboratory results, confirming myocardial amyloid deposition. This case supports the use of contrast adenosine stress echocardiography as a practical adjunct to advanced imaging for evaluating microvascular dysfunction.

The case presented by Yuting et al. underscores the central role of multimodality cardiovascular imaging in modern cardiology. In a 54-year-old woman with exertional dyspnea and signs of heart failure, multimodality imaging demonstrated apical hypertrophy with cavity obliteration, endocardial fibrosis, and calcification. ^18^F-FDG PET-CT showed intense apical uptake, initially raising concern for malignancy. However, CMR with late gadolinium enhancement (LGE) supported a diagnosis of hypertrophic cardiomyopathy. Endomyocardial biopsy and genetic testing further identified a pathogenic MYH7 variant, consistent with this diagnosis. The importance of CMR and genetic testing in hypertrophic cardiomyopathy has also been highlighted in recent large multicenter studies (4).

Another case underscoring the role of multimodality imaging with cardiac masses has been presented by Wang et al. Pericardial involvement with myxoid liposarcoma is exceptionally rare and usually indicates metastatic disease. In the present case multimodal imaging (CT, ultrasound, CMR) revealed a pericardial mass compatible with metastatic liposarcoma. The diagnosis was confirmed by histopathology and molecular testing. The role of CMR for the diagnostic classification and risk stratification of patients with cardiac masses has been recently demonstrated in single- and multi-center studies (5, 6).

In a related case with cardiac malignancy, Konuş presents a 47-year-old female suffering from heart failure induced by a giant primary pericardial schwannoma. Although multimodality imaging established the presence and extension of the cardiac tumor, the definitive preoperative histological diagnosis was achieved using a method rarely employed in cardiovascular diseases, namely endoscopic ultrasound-guided fine needle aspiration (EUS-FNA). This report supports the use of this method as an alternative to open surgical biopsy for lesions situated favorably relative to the esophageal tract.

The versatility of modern CMR allows the assessment of myocardial function, perfusion, viability and if required myocardial metabolism and tissue characterization and strain within a single examination and without radiation exposure for the patients (7, 8). However, extrasystoles may pose a significant limitation for image quality with CMR. This is highlighted in, where Röwer et al. report on the ability of real-time CMR to offer high image quality during free breathing and, in combination with an ECG provide a unique opportunity to analyze cardiac hemodynamics, which was not feasible using conventional CMR sequences.

### The utility of multimodality imaging in procedural guidance

In the report by Mandic et al., the authors present a 65-year-old female with severe hypertrophic obstructive cardiomyopathy diagnosed by multimodality imaging and referred for alcohol septal ablation. However, the presence of a rare congenital coronary artery anomaly with a single right coronary artery led to procedural failure, thus prompting the operators to use an alternative approach with micro-coil embolization. CT angiography visualized the complex anatomy and the successful closure of the septal branch which was accompanied by substantial reduction of the gradient and improvement in the clinical status. This case supports the use of micro-coil embolization as a viable, localized alternative when hostile anatomy precludes standard alcohol delivery.

Also supporting the value of an integrative multimodality imaging approach for interventional guidance, Karanasos et al. describe a rare case of an iatrogenic left main (LM) to left ventricle coronarocameral fistula discovered a decade after surgical aortic valve replacement. The integration of coronary angiography, three-dimensional transesophageal echocardiography and intravascular ultrasound (IVUS) was used to differentiate a true fistulous tract from paravalvular regurgitation. Percutaneous coronary intervention of the severely calcified LM disease was prioritized, thereby securing epicardial flow and deferring the high-risk closure of the fistula itself. Importantly, the procedure was guided by IVUS, consistent with the pivotal role of intravascular imaging in this lesion subset (9).

Li et al. describe a case where angiographic guidance was preferred over traditional surgical drainage for the management of a challenging loculated, eccentric purulent pericarditis in a 60-year-old male. Using a coronary guidewire and a microcatheter, the operators safely accessed the pericardial space for drainage and alteplase administration. This off-label use of traditional coronary intervention tools provided an alternative for minimizing surgical risk in a highly comorbid patient. However, this approach should be cautiously applied as there is a risk of iatrogenic myocardial injury when utilizing off-label coronary wires in inflamed pericardial spaces.

### Diagnostic pitfalls and complications of imaging procedures

Several cases in this collection serve to highlight several limitations of cardiovascular imaging and a need for careful interpretation always considering the clinical presentation. Chen et al. present two cases with localized right sinus of Valsalva dissection masquerading clinically and electrocardiographically as inferior STEMI due to extrinsic right coronary artery compression. In these cases, high clinical suspicion prompted the authors to use additional imaging, i.e., aortic root angiography and CT angiography in one case and IVUS in the other to establish the diagnosis of localized right sinus of Valsalva dissection. Similarly, Jia et al. report on two patients with localized aortic root dissection that initially evaded detection. Both patients had atypical symptoms and severe aortic regurgitation, and despite a multimodality imaging approach including transthoracic and transesophageal echocardiography and CT angiography, were misclassified as congenital supravalvular aortic membranes, with the correct diagnosis of localized aortic root dissection being established in the operating room and supported by retrospective analysis of the CT angiography images. The manuscript tells a cautionary tale regarding the diagnostic pitfalls inherent in evaluating severe aortic regurgitation of unclear etiology, particularly when patients lack classical symptoms. Furthermore, it is important to acknowledge that in such cases transesophageal echocardiography has a complementary role for guidance in the operating room (Malaguti et al.) (10).

Cai et al. highlight the potential risks and complications of invasive diagnostic procedures. A 62-year-old woman with arterial hypertension underwent diagnostic coronary angiography using 40 mL of the iso-osmolar contrast medium iodixanol. Ten minutes later, she developed rapidly progressive bilateral visual loss that progressed to complete blindness. Emergent cranial CT and MRI showed no evidence of acute infarction or hemorrhage, and contrast-induced transient cortical blindness was suspected. With supportive treatment, her vision returned to normal within one week. This case supports the initial use of non-invasive imaging, as recommended by current position statements and guidelines (11).

### Cardiovascular imaging in uncommon clinical scenarios

Our case collection further includes several cases showcasing the role of imaging in diagnosing congenital disorders of the heart and pulmonary circulation (Hattab et al.; Junqi et al.; Yang et al.; Wang; Qin et al.; Yana and Marat), or other rare conditions such as pneumopericardium (Wang et al.), or Takotsubo myocardiopathy (Teng et al.), while the case of Huang et al. shows how imaging data can be used in biomechanical analysis to develop predictive models for aneurysm rupture.

Overall, this case report collection highlights the growing role of advances in non-invasive cardiac imaging for diagnostic classification and risk stratification in patients with cardiac diseases. Integrating these modalities into routine clinical practice, particularly in challenging cases such as those presented here, can support timely and precise diagnosis, improve patient management, and ultimately improve patient management and outcomes.
